# UV-Irradiation- and Inflammation-Induced Skin Barrier Dysfunction Is Associated with the Expression of Olfactory Receptor Genes in Human Keratinocytes

**DOI:** 10.3390/ijms22062799

**Published:** 2021-03-10

**Authors:** Wesuk Kang, Bomin Son, Soyoon Park, Dabin Choi, Taesun Park

**Affiliations:** Department of Food and Nutrition, BK21 FOUR, Yonsei University, 50 Yonsei-ro, Seodaemun-gu, Seoul 120-749, Korea; wesuk42@naver.com (W.K.); mim1110@naver.com (B.S.); thdbs1201@naver.com (S.P.); vin1411@naver.com (D.C.)

**Keywords:** olfactory receptor, skin barrier, keratinocytes, barrier dysfunction

## Abstract

Olfactory receptors (ORs) have diverse physiological roles in various cell types, beyond their function as odorant sensors in the olfactory epithelium. These previous findings have suggested that ORs could be diagnostic markers and promising therapeutic targets in several pathological conditions. In the current study, we sought to characterize the changes in the expression of ORs in the HaCaT human keratinocytes cell line exposed to ultraviolet (UV) light or inflammation, well-recognized stimulus for skin barrier disruption. We confirmed that major olfactory signaling components, including ORs, *GNAL*, *Ric8b*, and adenylate cyclase type 3, are highly expressed in HaCaT cells. We have also demonstrated that the 12 ectopic ORs detectable in HaCaT cells are more highly expressed in UV-irradiated or inflamed conditions than in normal conditions. We further assessed the specific OR-mediated biological responses of HaCaT cells in the presence of known odorant ligands of ORs and observed that specific ligand-activated ORs downregulate skin barrier genes in HaCaT cells. This study shows the potential of OR as a marker for skin barrier abnormalities. Further research is needed to explore how OR is implicated in the development and progression of barrier dysfunction.

## 1. Introduction

Skin is the largest human organ, which acts as a barrier between the body and environmental conditions, thereby blocking pathogen entry and preventing physical and chemical effects as well as excessive water and solute loss. These functions are due to the physical properties of the stratum corneum (SC) that comprises differentiated keratinocytes (corneocytes) [1,2,3]. At the late stages of keratinocyte differentiation, soluble proteins, such as filaggrin, develop a scaffold on the cellular membrane, along with the supra-basal keratins (e.g., keratin 1 and keratin 10). Thereafter, other barrier-related proteins, including loricrin, are attached to this scaffold, completing the corneocyte backbone with lipids [3,4,5]. Notably, numerous studies have documented that ultraviolet (UV) light and inflammation are the major causes for disruption of the epidermal barrier function, accompanied by decrease in keratin, filaggrin, and involucrin levels. Impaired skin barrier function promotes percutaneous sensitization and results in a vicious cycle between barrier dysfunction and skin inflammation [6,7,8,9].

Olfactory receptors (ORs) form the largest subfamily of G-protein-coupled receptors and were previously thought to be exclusively present in the olfactory epithelium. Nevertheless, recent studies have revealed that ORs are more versatile than originally thought: they are emerging as general chemoreceptors found in diverse tissues (e.g., skin, liver, muscle, and prostate) [10,11,12], and their expression is associated with several pathological conditions [13,14,15,16,17,18,19,20]. For example, OR family 2 subfamily AG member 2 (OR2AG2) expression is significantly lower in the lung tissues of patients with asthma than that in those of healthy subjects [21]. In addition, OR10H1 gene expression is significantly higher in bladder cancer tissues than in normal bladder tissue, and the stimulation of this receptor by its ligand suppresses bladder cancer progression [22]. These findings suggest ORs to be diagnostic markers and promising therapeutic targets in several diseases, such as cancer and metabolic disorders. Furthermore, two ORs (OR10G7 and OR2AT4), which are expressed in human keratinocytes, were reported to be related to atopic dermatitis and wound healing processes, respectively [23,24].

Skin barrier dysfunction is closely linked to various dermatological disorders, including psoriasis and atopic dermatitis, resulting in erythema, itching, and pain [25,26]. The diagnosis and treatment of skin barrier dysfunction are urgent matters, but the factors involved in this pathological condition are not fully established. In this study, we aimed to characterize the changes in OR expression in human keratinocytes exposed to UV light or inflammation, well-recognized stimuli for skin barrier disruption. We further assessed the specific OR-mediated biological responses of human keratinocytes in the presence of known odorant ligands of the ORs.

## 2. Results

### 2.1. Skin Barrier Genes Are Downregulated in UVB-Irradiated HaCaT Cells

**Figure 1:**
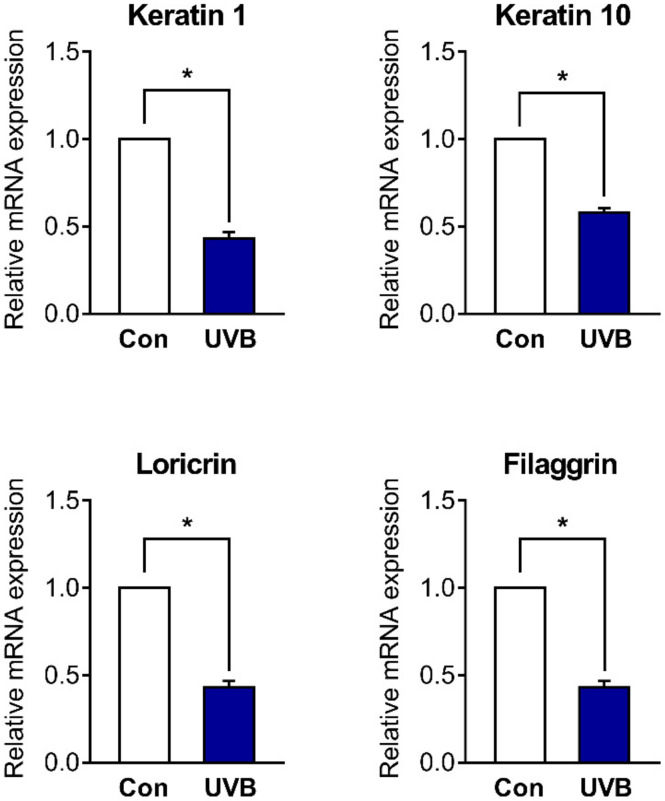
Skin barrier genes are downregulated in ultraviolet B (UVB)-irradiated HaCaT cells. The cells were treated with or without UVB irradiation (10 mJ/cm^2^) and harvested after 48 h. Then, the relative mRNA expression of skin barrier genes (*keratin 1*, *keratin 10*, *loricrin,* and *filaggrin*) was determined. Results are shown as mean ± standard error of the mean (SEM) of three experiments. * *p* < 0.05, between the groups.

In our preliminary study, we found that treatment of HaCaT cells with different doses of UVB (5, 10, 20, and 40 mJ/cm^2^) induced cell cytotoxicity in a dose-dependent manner. On the other hand, the expression of filaggrin was significantly downregulated over 10 mJ/cm^2^ (Supplementary Figure S1). We therefore decided to use UVB at a dose of 10 mJ/cm^2^ for all subsequent experiments, which can induce skin barrier dysfunction with minimum cell toxicity. We evaluated whether UVB irradiation in HaCaT cells decreased the expression of skin barrier genes, such as *keratin 1*, *keratin 10* and *loricrin*. The mRNA expression of all skin barrier genes was significantly decreased 48 h after UVB irradiation (Figure 1).

### 2.2. Skin Barrier Genes Are Downregulated in Inflamed HaCaT Cells

**Figure 2:**
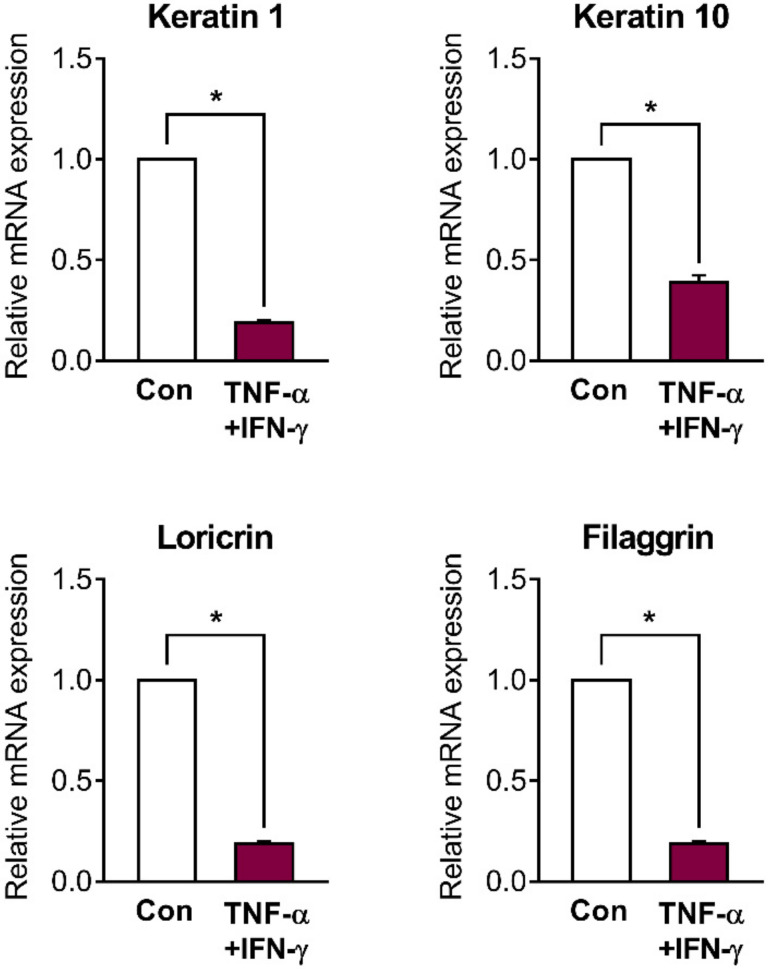
Skin barrier genes are downregulated in inflamed HaCaT cells. The cells were stimulated with or without tumor necrosis factor (TNF)-α/interferon (IFN)-γ (10 ng/mL each) for 48 h. Then, the cells were harvested and the relative mRNA expression of skin barrier genes (*keratin 1*, *keratin 10*, *loricrin,* and *filaggrin*) was measured. Results are shown as mean ± SEM of three experiments. * *p* < 0.05, between the groups.

A number of previous studies, including our own, have demonstrated that treatment of tumor necrosis factor (TNF)-α/interferon (IFN)-γ at a concentration of 10 ng/mL each is enough to induce inflammatory reactions, including increased cytokine levels, in HaCaT cells [27,28,29,30,31]. Therefore, all experiments using TNF-α and IFN-γ were performed at a concentration of 10 ng/mL each. We investigated whether the TNF-α/ IFN-γ-induced inflammation in HaCaT cells decreased the expression of skin barrier genes such as *keratin 1*, *keratin 10*, *loricrin*, and *filaggrin*. Similarly to that after UVB irradiation, the mRNA expression of all skin barrier genes was significantly downregulated 48 h after TNF-α/IFN-γ treatment (Figure 2).

### 2.3. Presence of Olfactory Signaling Pathway Components in HaCaT Cells

**Figure 3:**
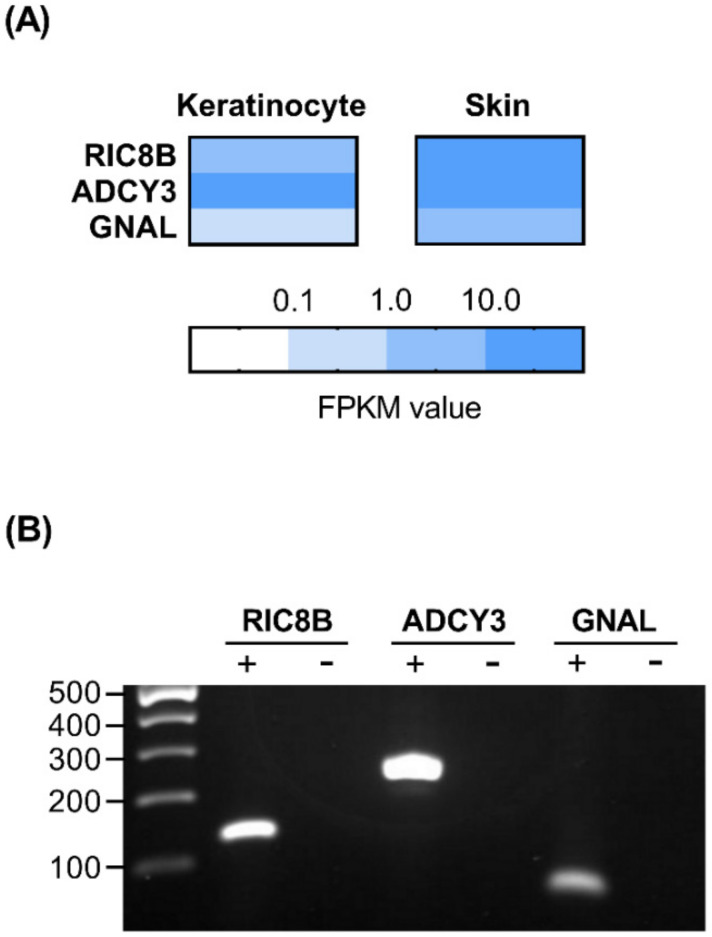
Presence of olfactory signaling pathway components in HaCaT cells. (**A**) Next-generation sequencing data of HaCaT cells and human skin tissues from the genotype-tissue expression (GTEx) database revealed the presence of three olfactory signaling pathway components genes, including *Ric8b*, adenylate cyclase type 3 (*ADCY3)*, and G Protein Subunit Alpha L (*GNAL)*. Dark blue represents high transcript expression, and white indicates low to no detectable transcript expression. (**B**) Reverse transcription polymerase chain reaction (RT-PCR) for *Ric8b*, *ADCY3*, and *GNAL* verification in HaCaT cells. The mRNA samples converted into cDNA are shown as (+), and the negative controls containing mRNA are shown as (-). Numbers, length of fragments (bp).

We determined whether the major components of the olfactory signaling pathway exist in HaCaT cells. To investigate the gene expression profile of HaCaT cells, RNA-Seq data were generated using next-generation sequencing (NGS), and the quantitative expression values were calculated for each sample based on the number of fragments per kilobase of exon per million fragments mapped (FPKM). These data have been deposited in the NCBI Bioproject PRJNA692826 (BioSample accession number SAMN17371264– SAMN17371266). Genes, such as *Ric8b*, adenylate cyclase type 3 (*ADCY3)*, and G Protein Subunit Alpha L (*GNAL)*, which are known to be involved in olfactory signal transduction, were expressed in HaCaT cells, as revealed from the NGS data using a colored scale. Additionally, we used the genotype-tissue expression (GTEx) database to identify that these genes are present in human skin (Figure 3A) [32]. We also verified the mRNA expression of these genes in HaCaT cells using semi-quantitative reverse transcription polymerase chain reaction (RT-PCR). The bands were detected in cDNA samples, which are shown as (+); on the contrary, no bands were observed in negative controls containing RNA samples, which are shown as (−) (Figure 3B). Results on the expression of such components may hint at the existence of upstream olfactory signal transduction elements, such as ORs, in HaCaT cells.

### 2.4. OR Profile in HaCaT Cells

**Figure 4:**
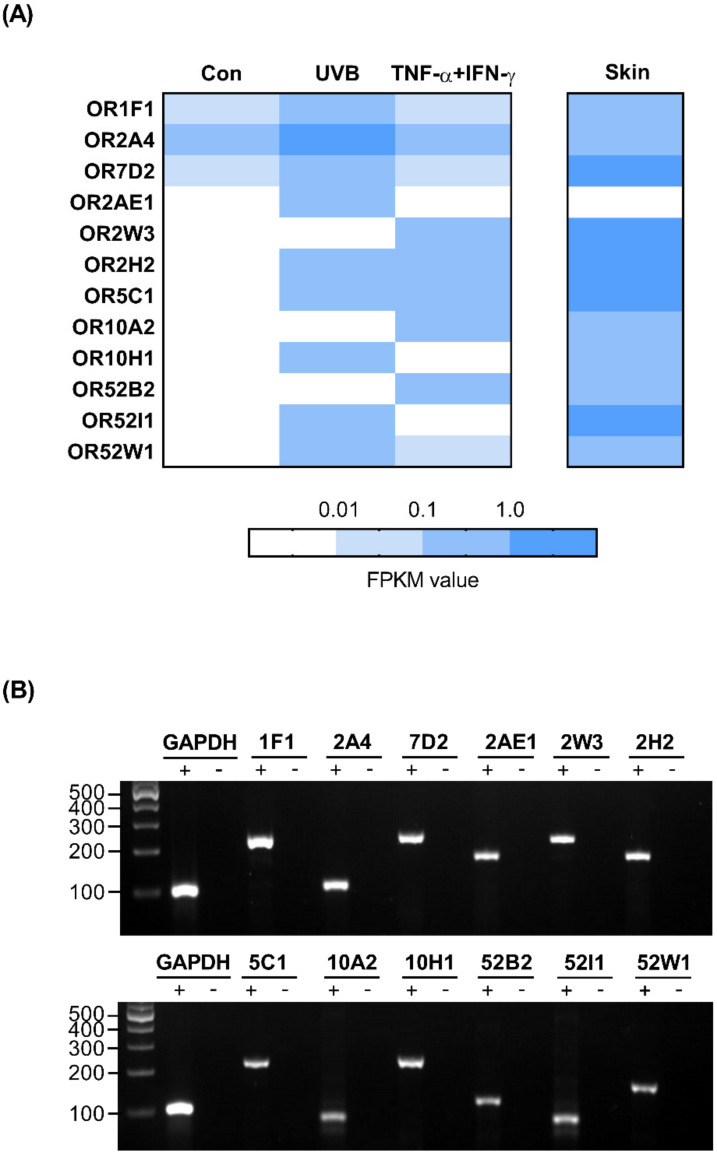
Olfactory receptor (OR) profile in HaCaT cells. (**A**) The heat map shows fragments per kilobase of exon per million fragments mapped (FPKM) values for the 12 highly expressed ORs found in the HaCaT cells and skin tissues. Dark blue represents high transcript expression, and white indicates low to no detectable transcript expression. (**B**) RT-PCR for verifying *OR1F1*, *OR2A4, OR7D2, OR2AE1, OR2W3, OR2H2, OR5C1, OR10A2, OR10H1, OR52B2, OR52I1,* and *OR52W1* expression in HaCaT cells. The housekeeping gene glyceraldehyde 3-phosphate dehydrogenase (*GAPDH*) was used for cDNA quality control. The mRNA samples converted into cDNA are shown as (+), and the negative controls containing mRNA are shown as (−). Numbers, length of fragments (bp).

To profile the OR expression in a skin barrier-impaired HaCaT cell model, we investigated the RNA-Seq data from UVB-irradiated and inflamed, as well as untreated, HaCaT cells. We analyzed the expression of ORs in two different treatment groups compared with that in the untreated control group and found that 12 OR genes were upregulated, and surprisingly, no OR genes were downregulated. In addition, using data obtained from the GTEx database, we found that, except for OR2AE1, 11 ORs were also expressed in sun-exposed human skin (Figure 4A). To validate the NGS data of transcript expression, we confirmed the presence of cDNA bands of the above-mentioned OR genes, whereas no bands were detected in the negative controls (Figure 4B).

### 2.5. Investigation of OR Expression in UVB-Irradiated and Inflamed HaCaT Cells

**Figure 5:**
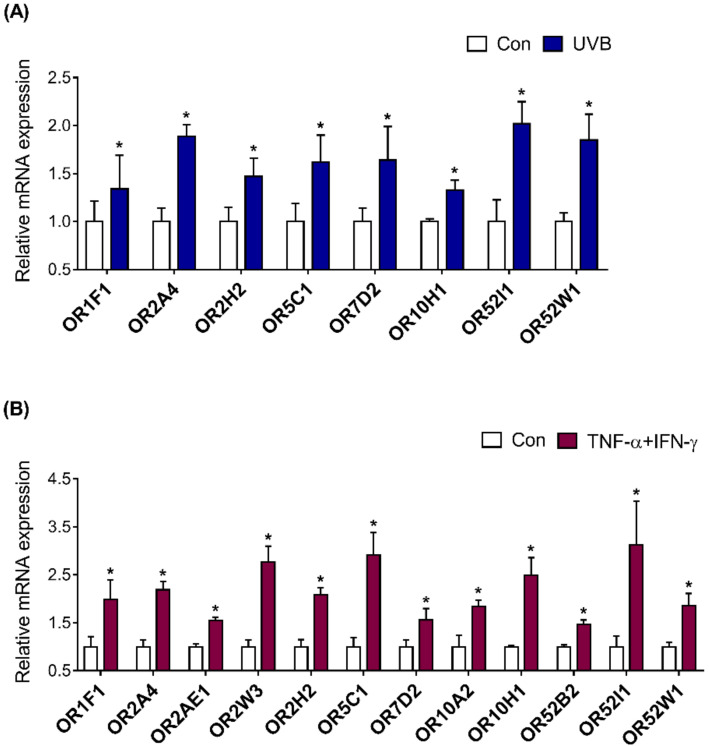
Investigation of olfactory receptor (OR) expression in UVB-irradiated and inflamed HaCaT cells. (**A**) The cells were treated with or without UVB irradiation (10 mJ/cm^2^) and incubated for 48 h. Then, the relative mRNA expression of eight ORs (*OR1F1, OR2A4, OR2H2, OR5C1, OR7D2, OR10H1, OR52I1,* and *OR52W1*) was determined. (**B**) HaCaT cells were stimulated with or without TNF-α/IFN-γ (10 ng/mL each) and harvested after 48 h. Thereafter, the relative mRNA expression of 12 ORs (*OR1F1, OR2A4, OR2AE1, OR2W3, OR2H2, OR5C1, OR7D2, OR10A2, OR10H1, OR52B2, OR52I1,* and *OR52W1*) was evaluated. Results are shown as mean ± SEM of three experiments. * *p* < 0.05, between the groups.

With the OR genes listed from NGS data, we further verified whether changes in the expression of these genes are associated with UVB-irradiated and inflamed HaCaT cells, using quantitative real-time PCR analysis. The values for the relative expression of 12 ORs were analyzed, and gene expression of eight ORs (OR1F1, OR2A4, OR2H2, OR5C1, OR7D2, OR10H1, OR52I1, and OR52W1) was found to be significantly increased by UVB-irradiated HaCaT cells in comparison to that in the untreated control HaCaT cells (Figure 5A). Moreover, all 12 ORs (OR1F1, OR2A4, OR2AE1, OR2W3, OR2H2, OR5C1, OR7D2, OR10A2, OR10H1, OR52B2, OR52I1, and OR52W1) were significantly upregulated in the inflamed HaCaT cells in comparison to those in the untreated control HaCaT cells (Figure 5B). We also found that UVB irradiation increased the expression of inflammatory genes, including TNF-α and IFN-γ, in HaCaT cells (Supplementary Figure S2).

### 2.6. OR Involvement in the Skin Barrier Disruption Pathway in HaCaT Cells

**Figure 6:**
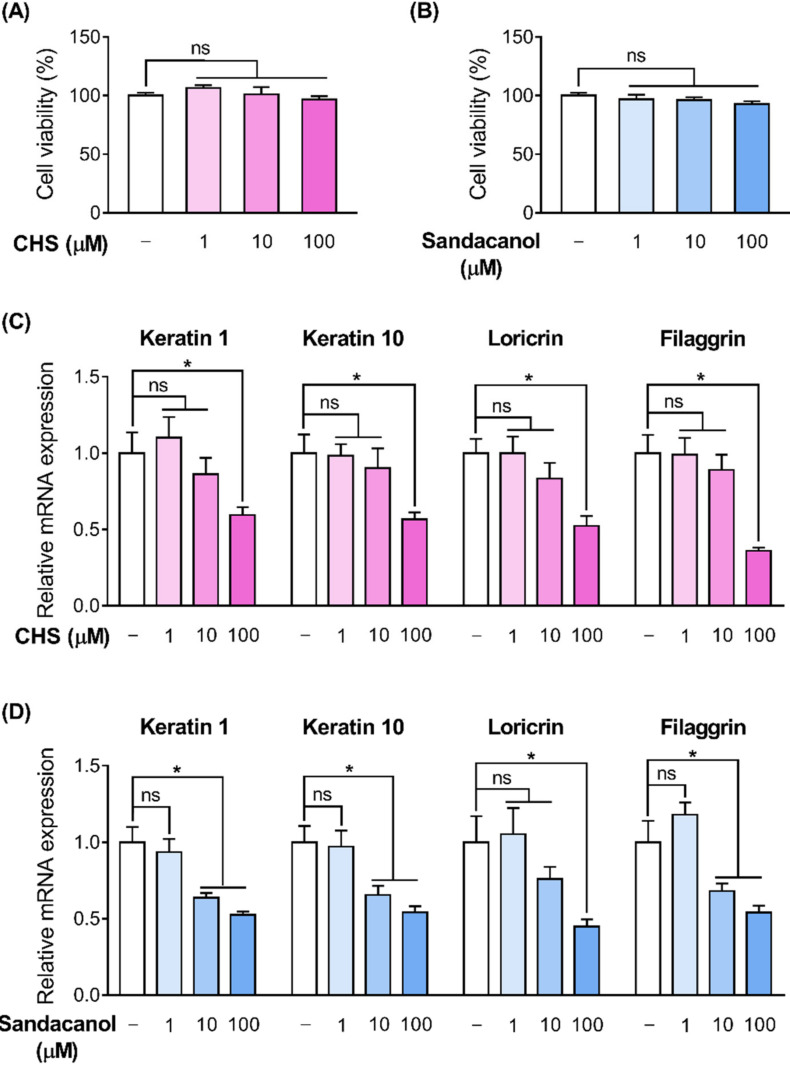
OR involvement in the skin barrier disruption pathway in HaCaT cells. The cells were cultured with vehicle, cyclohexyl salicylate (1, 10, or 100 µM), or sandacanol (1, 10, or 100 µM) for 48 h. Thereafter, (**A**,**B**) cell viability and (**C**,**D**) the relative mRNA expression of *keratin 1*, *keratin 10*, *filaggrin*, and *loricrin* were measured. Results are shown as mean ± SEM of three experiments. * *p* < 0.05, between the groups.

To explore whether the OR signaling pathway affects skin barrier disruption in HaCaT cells, we activated the ORs by treating HaCaT cells with their specific ligands and analyzed the expression level of *filaggrin,* a representative barrier marker. Unfortunately, the only known ligands of the ORs are cyclohexyl salicylate (CHS) and sandacanol for OR2A4 and OR10H1, respectively [22,33]. First, we confirmed that CHS and sandacanol (up to 100 μM each) did not affect the cell viability of HaCaT cells according to the water-soluble tetrazolium salt (WST-1) assay (Figure 6A,B). Then, we observed that the expression of *keratin 1*, *keratin 10*, *filaggrin*, and *loricrin* was downregulated after dose-dependent CHS treatment (1–100 μM) in HaCaT cells (Figure 6C). Similarly, the expression of these skin barrier genes was also downregulated after dose-dependent sandacanol treatment (1–100 μM) (Figure 6D).

## 3. Discussion

The OR superfamily has approximately 400 functional members in humans. Although virtually all ORs are expressed in the olfactory epithelium, the OR genes expressed in ectopic tissues are tissue- and condition-specific [23,34,35,36]. In this study, we confirmed that the expression of at least 12 ORs was significantly upregulated in UV-irradiated or inflamed keratinocytes. Among the 12 ORs, some ORs, such as OR2A4, OR2W3, OR2H2, OR7D2, OR10H1, and OR10A2, are expressed in at least three other tissue types, while the remaining, to the best of our knowledge, seem to be specifically expressed in skin [36]. Notably, the expression of all the OR genes, which was significantly increased by UVB irradiation, was also remarkably elevated by inflammation. Since UVB irradiation stimulated the inflammatory response, including the upregulation of TNF-α and IFN-γ levels, in this study, it is plausible to speculate that UV-mediated changes in OR expression might be associated with the inflammatory processes in human keratinocytes. Also, we conducted all experiments using the HaCaT cell line, a well-established in vitro differentiation model [37,38]. However, considering that characteristics of the HaCaT cell line are largely different from those of primary human keratinocytes, further research using primary keratinocytes is needed to further confirm that the phenomena observed in HaCaT cells are the common characteristics of human keratinocytes.

In the olfactory sensory neurons located in the nasal epithelium, OR activation by odorants or other stimuli can switch on a specific olfactory G-protein (*GNAL*), which in turn stimulates *Ric8b* and *ADCY3* and leads to cyclic adenosine monophosphate (cAMP) generation from adenosine triphosphate (ATP) [36,39,40]. Although the generated cAMP is primarily involved in transmitting odor information to the olfactory bulbs in the brain, it has been demonstrated that an increase in cellular cAMP by *ADCY3* could stimulate protein kinase A (PKA) signaling, which is well-known to regulate diverse physiological and pathological processes [41,42,43]. Notably, the distribution of olfactory transduction components is not limited to the olfactory sensory neurons. Several reports have demonstrated that some cells in some ectopic tissues constitutively express components of the olfactory transduction machinery such as ORs, Golf, *Ric8b*, and *ADCY3* [36,40,44]. Consistent with previous studies, we confirmed that major olfactory signaling components, including ORs, Golf, RICB8b, and *ADCY3,* are highly expressed in human keratinocytes. Thus, it is speculated that the activation of ectopically expressed ORs may target the cAMP–PKA pathways, thereby mediating various functions including skin barrier formation.

In this study, we first screened the OR genes whose expression was altered in response to UV and inflammation, using high-throughput NGS data. Next, we validated the expression of the screened 12 OR genes in human keratinocytes by using PCR. Notably, we observed that many ORs not detected with RNA-Seq were detected with PCR. Discrepancies between PCR and RNA-Seq data can be due to the higher sensitivity of PCR than that of the RNA-Seq. A previous study, using both RNA-Seq and PCR, reported that 22 specific ORs were found to be expressed in human colon tissues, using PCR; however, the expression of 12 of these was not detected in RNA-Seq datasets [36]. Therefore, the ORs detected with RNA-Seq analysis in this study may be considered as the minimum number of expressed ORs in human keratinocytes; however, we do not exclude the possibility that more ORs remained undetected using RNA-Seq analysis. Based on our preliminary study, ORs can certainly not yet be considered skin dysfunction markers that are powerful enough to replace already known markers such as filaggrin and involucrin, but ORs might be used in combination with known biomarkers in order to enhance the sensitivity of diagnosis of barrier dysfunction, or to predict if the subject would show skin barrier abnormalities in the future.

In the present experiments, TNF-α/IFN-γ were used among many inflammatory stimuli and UVB was chosen among UV rays for developing in vitro barrier dysfunction models, based on the previous studies on inflammatory stimuli and UV. To date, a total of more than 100 cytokines and chemokines have been described in mammals. Among them, TNF-α and IFN-γ have been generally accepted as primary inflammatory cytokines as they are produced during the early cellular response to inflammatory stimuli (e.g., allergens), which activates the cell towards the production of various secondary inflammatory cytokines, including interleukin (IL)-6, IL-8, IL-12, and IL-15, and chemokines such as adhesion molecules intercellular adhesion molecule-1 (ICAM-1), thymus and activation-regulated chemokine (TARC), and macrophage-derived chemokine (MDC). Thus, treatment of TNF-α and IFN-γ has been regarded as a common in vitro model for inflammatory skin disorders [9,45,46,47]. On the contrary, UV is classified by three wavebands: UVA (315–400 nm), UVB (280–315 nm), and UVC (100–280 nm). UVB is blocked by the ozone layer. Although sunlight contains approximately 20 times more UVA than UVB, it is widely accepted that UVB is the leading factor in UVB-irradiated skin damage owing to its high energy [48,49,50]. Numerous recent studies have shown that only UVB radiation is enough to adversely affect the epidermal barrier function, including permeability barrier disruption, increased trans-epidermal water loss, and decreased SC hydration [51,52,53,54]. In the current work, we confirmed that the treatment of TNF-α/IFN-γ and UVB successfully induced barrier dysfunction in HaCaT cells.

We observed that TNF-α/IFN-γ treatment and UVB irradiation upregulated several OR genes in keratinocytes. These increases may be functionally interpreted in two different ways: (i) OR receptors act as mediators of the events that initiate specific pathological conditions [23], and (ii) these receptors have protective functions and their expression is increased to compensate for unfavorable conditions [22,34]. To differentiate between two possible explanations for increased OR expression, we used a known, specific ligand to each receptor and investigated the receptor-mediated effects on the barrier function-related gene expression. Remarkably, we observed that the activation of OR2A4 and OR10H1 by their specific ligands, CHS and sandacanol respectively, downregulated *filaggrin* gene expression in human keratinocytes. We speculated that both OR2A4 and OR10H1 are involved in developing skin barrier dysfunction. Further functional characterization of ORs would enable the development of clinical applications for skin barrier dysfunction. If increased expression of ORs is found to lead to barrier dysfunction, these findings can encourage the development of compounds that antagonize the ORs. On the contrary, if increased expression of ORs is proven to have a protective function in the skin barrier, the ligands which specifically bind and activate these receptors may have great potential for clinical use in the treatment of skin barrier dysfunction.

## 4. Materials and Methods

### 4.1. Cell Culture

HaCaT keratinocytes were purchased from AddexBio Technologies (San Diego, CA, USA) and cultured at 37 °C in 5% CO_2_ in high-glucose Dulbecco modified Eagle’s medium (DMEM; HyClone, Logan, UT, USA) supplemented with 10% fetal bovine serum (FBS; Gibco, Grand Island, NY, USA) and 1% penicillin-streptomycin (Gibco). For developing in vitro barrier dysfunction models, TNF-α/IFN-γ treatment and UVB irradiation were used. First, 6 × 10^5^ HaCaT cells/well were seeded in six-well plates and cultured until confluence. Then, the cells were rinsed with PBS, covered with a thin PBS layer, exposed to 10 mJ/cm^2^ UVB irradiation for 10 s, and further incubated in DMEM for 48 h for developing the UVB-irradiated skin dysfunction models. A CL-1000M UV crosslinker (UVP; Upland, CA, USA), with a UV peak at 302 nm, was used for UVB irradiation. On the contrary, the medium was replaced with DMEM containing 10 ng/mL TNF-α or IFN-γ (R&D Systems; Minneapolis, MN, USA) and further cultured for 48 h for developing the inflamed skin dysfunction models. When required, the cells were treated with three different CHS (BOC sciences; Shirley, NY, USA) or sandacanol (Glentham Life Sciences; Wiltshire, UK) concentrations for 48 h.

### 4.2. WST-1 Assay

The WST-1 assay was conducted to evaluate cell viability. HaCaT cells were seeded in a 96-well plate at a density of 5000 cells per well and cultured until confluence. Then, UVB irradiation or the treatment of CHS or sandacanol was performed as described in Section 4.1. Next, the cells were further cultured for 3 h with the WST reagent (Sigma-Aldrich, Seoul, Korea), according to the manufacturer’s instructions. Absorbance was measured at 450 nm on the Infinite M200 microplate reader (Tecan, Grodig, Austria).

### 4.3. RNA Extraction and PCR

Total RNA was extracted from cells using the TRIzol reagent (Invitrogen, Carlsbad, CA, USA) and quantified on a NanoDrop spectrophotometer (Tecan). Thereafter, an equal amount of each RNA sample was subjected to cDNA synthesis using SuperScript IV Reverse Transcriptase (Invitrogen) according to the manufacturer’s instructions. To visually identify the specific genes expressed in keratinocytes, semi-quantitative PCR was conducted in a 20 μL solution, comprising 2× PCR MasterMix (Intron, Seoul, Korea), 0.6 μM of each primer, and 25 ng template, using the GeneMax thermal cycler (BIOER; Hanqzhou, China). Negative controls containing mRNA without reverse transcription were used to confirm the absence of DNA contamination and PCR specificity. For precise quantification, quantitative PCR was carried out in a 20 μL solution, comprising 10 μL SsoAdvanced Universal SYBR Green Supermix (Bio-Rad, Hercules, CA, USA), 0.6 μM of each primer, and 25 ng template cDNA, using the CFX Real-Time System (Bio-Rad). Glyceraldehyde 3-phosphate dehydrogenase (*GAPDH*) served as the housekeeping gene for gene expression normalization. Primer sequences used for each gene are listed in Supplementary Table S1.

### 4.4. RNA-Sequencing

Libraries were prepared from total RNA using the NEBNext Ultra II Directional RNA-Seq Kit (NEW ENGLAND BioLabs, Hitchin, UK). The mRNA was isolated using the above-mentioned methods for RNA extraction and PCR. The isolated mRNAs were prepared for cDNA synthesis and shearing, according to the manufacturer’s instructions, indexed using the Illumina indexes 1–12, and enriched using PCR. Thereafter, the libraries were checked with the Agilent 2100 Bioanalyzer (DNA High-Sensitivity Kit) to assess the mean fragment size. Quantification was performed using a library quantification kit and a StepOne Real-Time PCR System (Life Technologies Corporation, Carlsbad, CA, USA). High-throughput sequencing was carried out as paired-end 100 sequencing using HiSeq X10 (Illumina, San Diego, CA, USA). Quality control of raw sequencing data was conducted using FastQC [55]. Gene expression levels were estimated using FPKM values and normalized based on the quantile normalization method using EdgeR within R [56].

### 4.5. Statistical Analysis

Each set of experiments was performed independently in triplicate, and the results are expressed as mean ± standard error of the mean (SEM). Differences between groups were analyzed with the SPSS 25 software (SPSS, Chicago, IL, USA) using the unpaired Student’s *t*-test. *p* < 0.05 was considered statistically significant.

## 5. Conclusions

We have demonstrated that the specific ectopic ORs detectable in human keratinocytes are more highly expressed in barrier dysfunction conditions than in normal conditions. Furthermore, stimulating these ORs with their specific ligands altered the levels of skin barrier markers. The current study shows the potential of OR as a marker for skin barrier abnormalities. Further work is required to explore how OR is related to the development and progression of barrier dysfunction.

## Data Availability

All data generated or analyzed during this study are included in this published article.

## Supplementary Materials

The following are available online at https://www.mdpi.com/1422-0067/22/6/2799/s1. Figure S1: UVB irradiation affects cell viability and filaggrin gene expression in a dose-dependent man-ner in HaCaT cells, Figure S2: Inflammatory cytokine genes are upregulated in UVB-irradiated HaCaT cells, Table S1: Primer sequences.
