# Supplementary material for: UV-Irradiation- and Inflammation-Induced Skin Barrier Dysfunction Is Associated with the Expression of Olfactory Receptor Genes in Human Keratinocytes
Source: Int J Mol Sci. 2021 Mar 10;22(6):2799. doi: 10.3390/ijms22062799 (PMC7999531; doi:10.3390/ijms22062799)
Supplement: Supplementary file 1 [file ijms-22-02799-s001.zip › Supplementary Table 1..docx]

Supplementary Table 1. Primer sequences

| **Gene name** | **Primer sequences (5'→ 3')** |
| --- | --- |
| Keratin 1 | F: CCTTCTTCAGCCCCTCAATGTG  R: AGCAGCTCCCATTTTGTTTGC |
| Keratin 10 | F: CAACTCACATCAGGGGGAGC  R: CAGCTCATCCAGCACCCTAC |
| Loricrin | F: TCATGATGCTACCCGAGGTTTG  R: CAGAACTAGATGCAGCCGGAGA |
| Filaggrin | F: AGGCTCCTTCAGGCTACATTC  R: CAGGAGAGTAGACATCTTTTGGCA |
| Adenylate Cyclase 3 (*ADCY3*) | F: GCCCAACTTTGCTGACTTCTAC  R: GCTCTCTCTCGGACTTGTCTTC |
| RIC8 Guanine Nucleotide Exchange Factor B (*RIC8B*) | F: TGTGACGGTAGACAGTTGGAAG  R: GCATTGCTGTGTAGCTCTTCTG |
| G Protein Subunit Alpha L (*GNAL*) | F: CAGCAAGACGACGGAAGACC  R: CGCTCTTTCTGCAACTGCTTCTC |
| Olfactory Receptor Family 1 Subfamily F Member 1 (*OR1F1*) | F: ATGTATTTCGTTTTCATGTTCGTG  R: AGAAGTGAGTGATGGCATTGTCT |
| Olfactory Receptor Family 2 Subfamily A Member 4 (*OR2A4*) | F: TGGATACAGACCGTGAGGGA  R: ATAGCAGGAATGCCGATCCA |
| Olfactory Receptor Family 7 Subfamily D Member 2 (*OR7D2*) | F: TGCTGGGAAACCTGCTCATC  R: CATCACGGTCAGGAGTAGCG |
| Olfactory Receptor Family 2 Subfamily AE Member 1 (*OR2AE1*) | F: TTTGGTTTGGTGCCTGCATCTTC  R: AACATCTCTCCTCAGCACTCTTCTC |
| Olfactory Receptor Family 2 Subfamily W Member 3 (*OR2W3*) | F: CCTTGGCCATGTCTCCTGTGA  R: TCTGCCTTCCTGATGCTGACC |
| Olfactory Receptor Family 2 Subfamily H Member 2 (*OR2H2*) | F: CCATCTCACTGTGGTCACCCTCTTC  R: GAATGCCCTGGTTACCTCCTTGTTC |
| Olfactory Receptor Family 5 Subfamily C Member 1 (*OR5C1*) | F: TATCACGGTGTCTTATGGCTTCATC  R: GGCTGTAGATGAGTGGGTTGAG |
| Olfactory Receptor Family 10 Subfamily A Member 2 (*OR10A2*) | F: TTCTCTTCCCTGCCTACTGAAATAC  R: TCCCATCAGGGTGACCAGGTAG |
| Olfactory Receptor Family 10 Subfamily H Member 1 (*OR10H1*) | F: ATCTCCCTGTCACATCTCACC  R: GAACAGGTACATCAGCAGGAACAG |
| Olfactory Receptor Family 52 Subfamily B Member 2 (*OR52B2*) | F: AGAGAGCCAAAGCCCTCAATACC  R: TGTGGACAACCTCTGGCACATTC |
| Olfactory Receptor Family 52 Subfamily I Member 1 (*OR52I1*) | F: CACCATCAGAGCTGTCACATTCA  R: ACAACCACATTGGAGCCACAGA |
| Olfactory Receptor Family 52 Subfamily W Member 1 (*OR52W1*) | F: CTGACTGGCTTTCCAGGGCTA  R: GCCAGGATGGCCAACAGTAGA |
| Glyceraldehyde 3-phosphate dehydrogenase (*GAPDH*) | F: ATCAAGAAGGTGGTGAAGCAG  R: GTCGCTGTTGAAGTCAGAGG |
