# Supplementary material for: UV-Irradiation- and Inflammation-Induced Skin Barrier Dysfunction Is Associated with the Expression of Olfactory Receptor Genes in Human Keratinocytes
Source: Int J Mol Sci. 2021 Mar 10;22(6):2799. doi: 10.3390/ijms22062799 (PMC7999531; doi:10.3390/ijms22062799)
Supplement: Supplementary file 1 [file ijms-22-02799-s001.zip › Supplementary figures.docx]

**
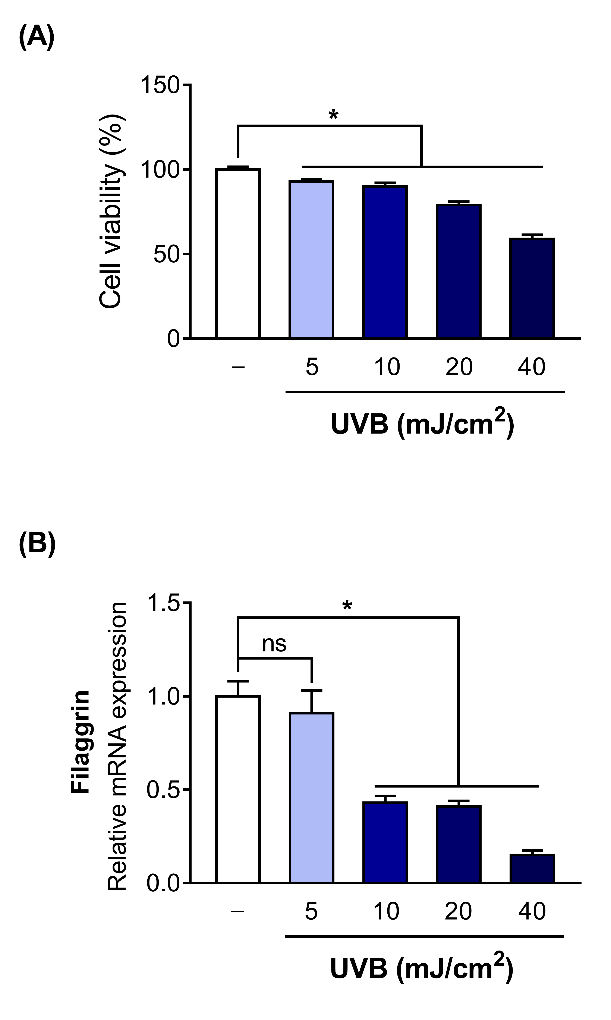
**

Figure S1. UVB irradiation affects cell viability and filaggrin gene expression in a dose-dependent manner in HaCaT cells. The cells were treated with or without UVB irradiation (5, 10, 20, and 40 mJ/cm^2^) and incubated for 48 h. Thereafter, (A) cell viability and (B) the relative mRNA expression of *filaggrin* were measured. Results are shown as mean ± SEM of three experiments. * p < 0.05, between the groups.

**
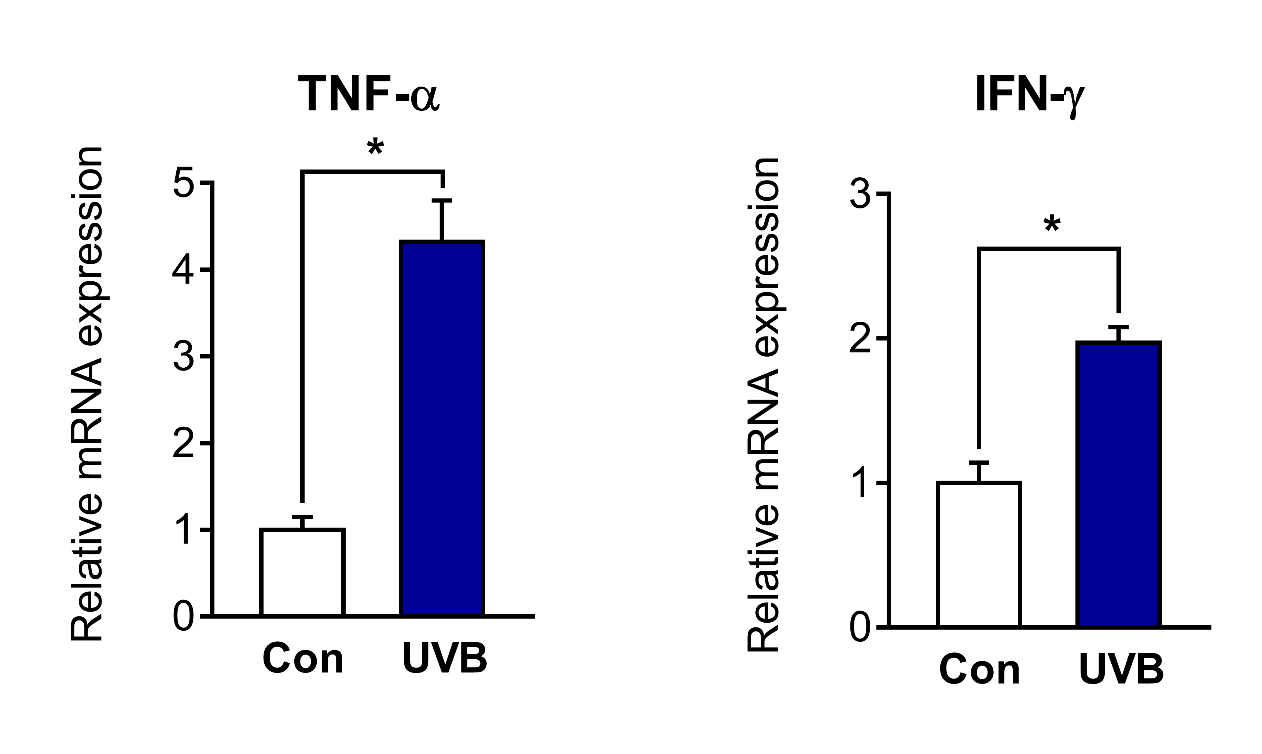
**

Figure S2. Inflammatory cytokine genes are upregulated in UVB-irradiated HaCaT cells. The cells were treated with or without UVB irradiation (10 mJ/cm^2^) and incubated for 48 h. Then, the relative mRNA expression of inflammatory cytokine genes (TNF-α and IFN-γ) was determined. Results are shown as mean ± SEM of three experiments. * p < 0.05, between the groups.
