# Supplementary figures and images for: UV-Irradiation- and Inflammation-Induced Skin Barrier Dysfunction Is Associated with the Expression of Olfactory Receptor Genes in Human Keratinocytes
Source: Int J Mol Sci. 2021 Mar 10;22(6):2799. doi: 10.3390/ijms22062799 (PMC7999531; doi:10.3390/ijms22062799)

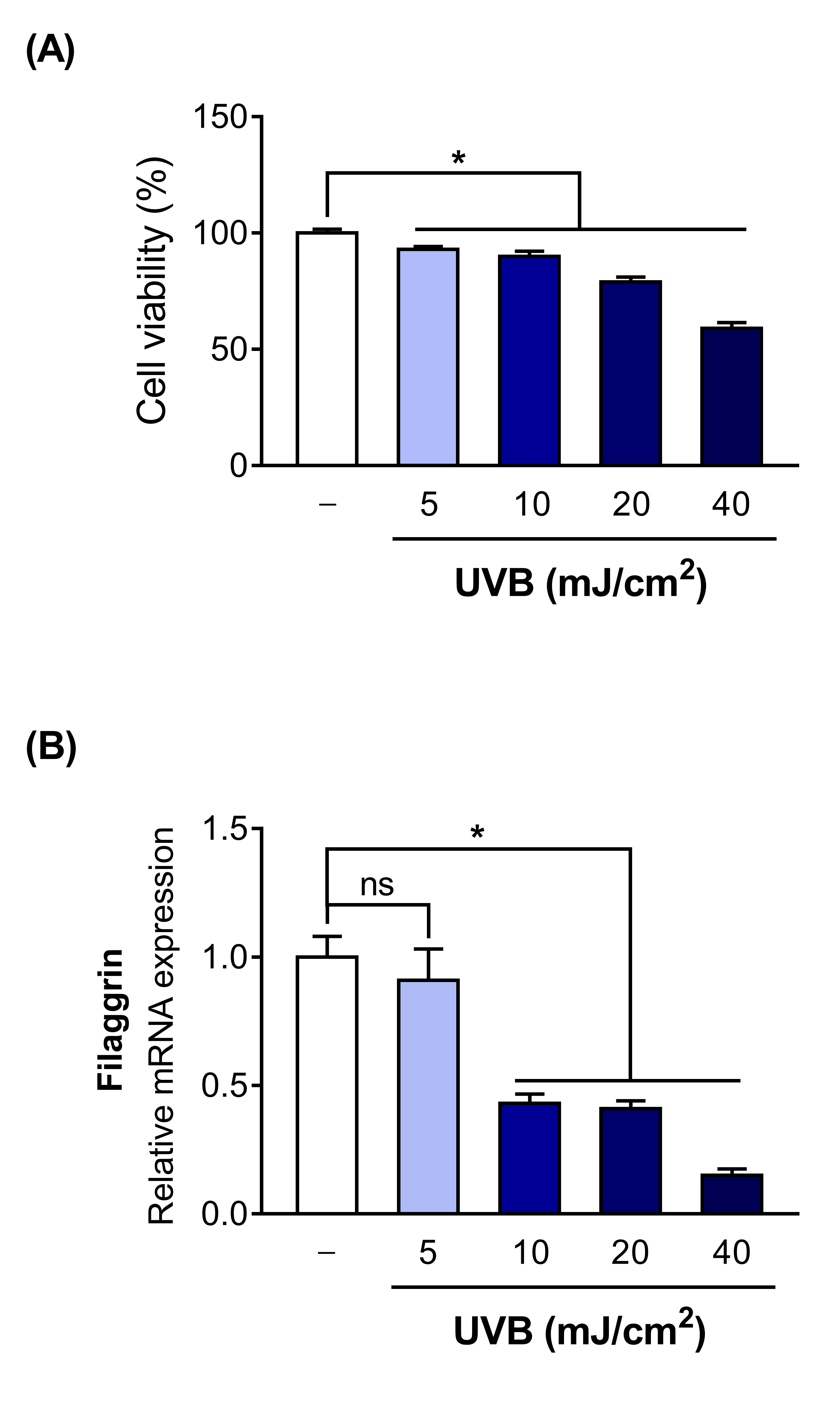

Supplement: Supplementary file 1 [file ijms-22-02799-s001.zip › Fig. S1.tif]

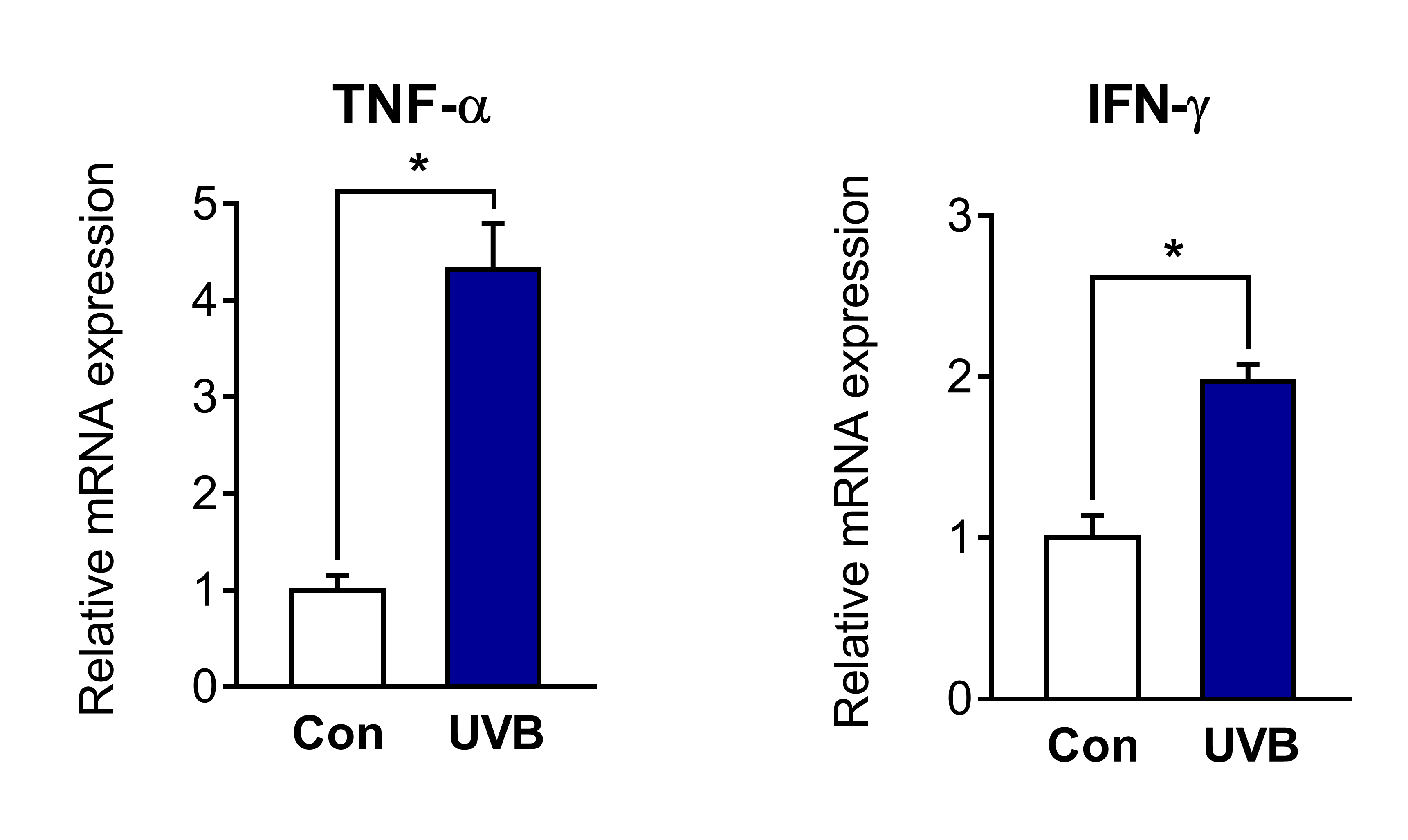

Supplement: Supplementary file 1 [file ijms-22-02799-s001.zip › Fig. S2.tif]
